# Novel Immune Cell-Based Therapies to Eradicate High-Risk Acute Myeloid Leukemia

**DOI:** 10.3389/fimmu.2021.695051

**Published:** 2021-08-03

**Authors:** Roberto Limongello, Andrea Marra, Antonella Mancusi, Samanta Bonato, Eni Hoxha, Loredana Ruggeri, Susanta Hui, Andrea Velardi, Antonio Pierini

**Affiliations:** ^1^Institute of Hematology and Centre of Haemato-Oncology Research (CREO), University and Hospital of Perugia, Perugia, Italy; ^2^Department of Radiation Oncology, City of Hope Medical Center, Duarte, CA, United States; ^3^Beckman Research Institute of City of Hope, Duarte, CA, United States

**Keywords:** HR-AML, poor outcome, adoptive immune therapies, CAR-T, HSCT, Treg-Tcon

## Abstract

Adverse genetic risk acute myeloid leukemia (AML) includes a wide range of clinical-pathological entities with extremely poor outcomes; thus, novel therapeutic approaches are needed. Promising results achieved by engineered chimeric antigen receptor (CAR) T cells in other blood neoplasms have paved the way for the development of immune cell-based therapies for adverse genetic risk AML. Among these, adoptive cell immunotherapies with single/multiple CAR-T cells, CAR-natural killer (NK) cells, cytokine-induced killer cells (CIK), and NK cells are subjects of ongoing clinical trials. On the other hand, allogeneic hematopoietic stem cell transplantation (allo-HSCT) still represents the only curative option for adverse genetic risk AML patients. Unfortunately, high relapse rates (above 50%) and associated dismal outcomes (reported survival ~10–20%) even question the role of current allo-HSCT protocols and emphasize the urgency of adopting novel effective transplant strategies. We have recently demonstrated that haploidentical allo-HSCT combined with regulatory and conventional T cells adoptive immunotherapy (Treg-Tcon haplo-HSCT) is able to overcome disease-intrinsic chemoresistance, prevent leukemia-relapse, and improve survival of adverse genetic risk AML patients. In this *Perspective*, we briefly review the recent advancements with immune cell-based strategies against adverse genetic risk AML and discuss how such approaches could favorably impact on patients’ outcomes.

## Introduction

High risk (or adverse risk) acute myeloid leukemias (HR-AML) include a number of clinical and biological AML subsets which are usually characterized by poor response to conventional treatments and dismal long-term survival, even when conventional allogeneic hematopoietic stem cell transplantation (allo-HSCT) is performed (1). Such AML category is characterized by high-risk cytogenetics [i.e., complex and/or monosomal karyotypes, chromosomes 3, 5, 7, and 17 aberrations) and/or by specific genetic signatures (including mutations in *TP53, RUNX1, ASXL1*, and *FLT3* genes (2)] that confer an aggressive phenotype and often chemoresistance. Moreover, a large proportion of patients affected by secondary AML (sAML) (3) and therapy-related leukemias (tr-AML) (4) converge into the HR-AML category. sAML is characterized by distinct molecular features, frequently involving the aberrant displacement of spliceosomal machinery (*SRSF2, SF3B1, U2AF1*, and *ZRSR2*), epigenetic modifiers (*ASXL1, EZH2, BCOR, RUNX1*), and cell-cycle regulators (*TP53*) (2). Despite the fact that next-generation sequencing (NGS) analyses have recently shed some light on the genetic complexity of these AML subsets, deep knowledge on leukemogenesis of each specific biological entity is currently lacking. Thus, targeted therapeutic approaches are still missing. While several drugs have been recently approved for the treatment of adult AML, they have only shown to slightly influence the fatal course of HR-AML patients. Such expanding *armamentarium* includes small molecules (e.g., FLT3 inhibitors, Midostaurin and Gilteritinib; isocitrate-dehydrogenase type 1 and 2/IDH1-2 inhibitors, Ivosidenib and Enasidenib; the Bcl2-inhibitor, Venetoclax) and new-generation cytotoxic treatments, like CPX-351 (5). Indeed, CPX-351 received Food and Drug Administration (FDA) 2019 approval for the treatment of tr-AML or AML with myelodysplasia-related changes (AML-MRC). Furthermore, emerging tailored strategies against mutant *TP53* (i.e., APR-246, Pevonedistat) (6–9) are providing encouraging yet preliminary evidences that may support their use in this high-risk setting. Since the achievement of durable remissions and the prevention of disease relapse remain major issues in the treatment of these patients, many research efforts have been directed towards a deeper understanding of mechanisms regulating relapse biology, with a major focus on immune system perturbation.

**Figure 1:**
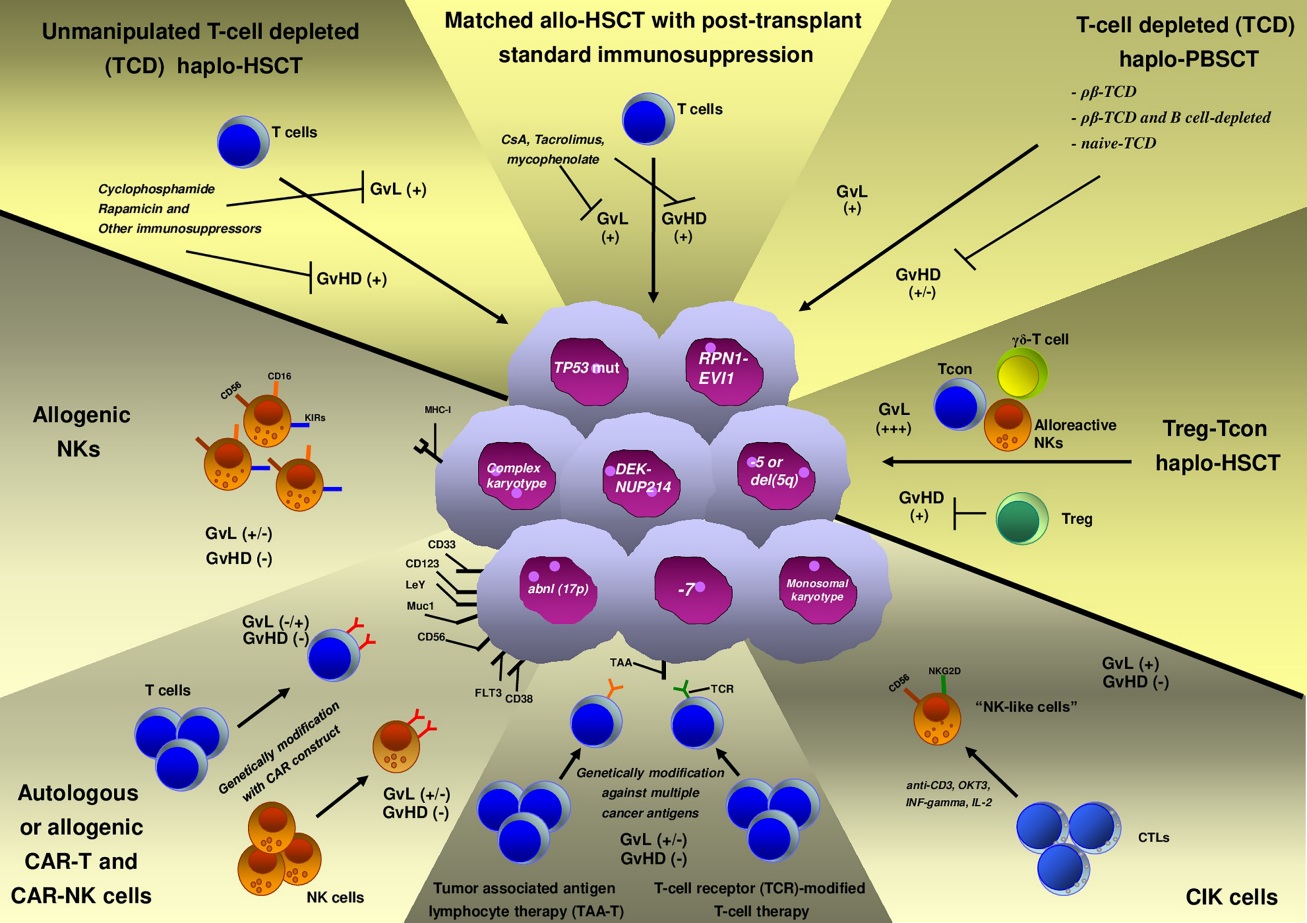
Available immune cell-based therapies for high-risk acute myeloid leukemia (HR-AML).

Immune-based adoptive cell therapies (ACTs) rely on the infusion of immune cells that aim to kill the tumor. These therapeutic platforms are revolutionizing treatment of blood neoplasms (**Figure 1**) and are challenging traditional drug interventions (10). In recent years, important advances have been made in developing novel effective immunotherapies (immune-checkpoint blockade, ACT, and vaccines) to overcome tumor-induced T-cell exhaustion and immune escape (10). Chimeric antigen receptor T-cells (CAR-T cells) are a form of ACT that has already demonstrated to be an effective treatment of various aggressive cancers, including subsets of advanced leukemias (11–13). Beyond this, a plethora of other immune cell-based approaches are currently under investigation in blood tumors, including CAR-natural killer (NK) cells, cytokine-induced killer cells (CIK), and NK cells, as well as novel forms of CAR-T cells (dual CAR-T and multi-CAR-T cells).

Allo-HSCT and especially HLA haploidentical allo-HSCT (haplo-HSCT) may serve as “discovery platforms” that can help to reveal the complex interplay between AML and the immune microenvironment and to set the base for pioneering studies of AML immune-targeting (**Figure 1**). However, conventional transplantation strategies have limited impact on HR-AML outcomes, as survival curves rarely exceed 30–35% (14–16). In order to improve such outcomes, novel allo-HSCT strategies that exert more potent antileukemic activity need to be developed. Adoptive immunotherapy with conventional T cells (Tcons) and regulatory T cells (Tregs) is an innovative strategy that has been built to overcome disease chemoresistance and boost T-cell immunity, while preserving host tissues from graft-*versus*-host disease (GvHD) damaging (17). In particular, Treg infusion in the absence of other forms of immune suppression allows for T cell-mediated killing of leukemic blasts. Thus, such approach resulted in prolonged and stable disease remission in the vast majority of HR-AML patients, as we have preliminarily observed in a *proof-of-concept* retrospective study (18).

In the present *Perspective*, we will review molecular mechanisms underlying HR-AML biology that drive disease relapse, as well as the potential impact of the newly developed approaches with target therapies on patients’ outcomes. We will describe immune-based strategies against HR-AML in ongoing trials and discuss how refined transplantation approaches with adoptive immunotherapy might represent the “ultimate” therapeutic option for a definitive eradication of HR-AML.

### Molecular Genetics of HR-AML

HR-AML includes many distinct biological entities, often characterized by an aggressive phenotype and intrinsic resistance to conventional treatments (2). HR-AML are not well defined in World Health Organization (WHO) classification, but this definition is widely used in clinical risk-adapted algorithms and also in the evaluation of the results from clinical trials. The major subgroup within this category consists of *AML with high-risk cytogenetics*, a subset of AML with different pathologic and clinical features that include the following:

- AML with complex karyotype (CK): its definition is not clear yet; however, it might be identified by the presence of ≥3 chromosomal abnormalities not included in defined WHO categories, and not associated with favorable prognosis (2, 19, 20)
- AML with monosomal karyotype (MK): it is defined by the presence of at least two autosomal monosomies or one single autosomal monosomy in combination with at least one structural abnormality (21)
- AML bearing specific chromosomal aberrations: it is defined by the presence of specific genetic abnormalities such as inv(3)(q21.3q26.2) or t(3;3)(q21.3;q26.2); *GATA2,MECOM(EVI1)*; -5 or del(5q); -7; -17/abn(17p) (2)

Specifically, CK AML have been recently proposed to be further divided into *typical* and *atypical* cases by the presence (or the absence, respectively) of 5q, 7q, and/or 17p losses (22). *Typical* CK AML category bears *TP53* mutations (almost absent within the *atypical* subgroup) more frequently and, thus, it is associated with poorer outcomes (compared to *atypical* cases). On the other hand, *atypical* CK AMLs are characterized by different mutational onco-prints and more frequently display mutations of RAS pathway–associated genes, *NPM1* and/or *FLT3* genes (22). Functional transcriptomic analyses of CK AML highlighted an elevated genomic instability with aberrant activation of DNA damage response and cell-cycle checkpoint pathways (23, 24). Although at very initial stages, genomic analyses of MK AML cases have consistently demonstrated that abnormalities involving chromosomes 5 [-5, or del(5q)], 7 (-7), and 17 (-17/abn(17p) are frequent in this setting, and strongly coupled to *TP53* pathogenic mutations (25). However, other molecular pathways are implicated in MK AML pathogenesis and rely on a peculiar mutational signature targeting *NOTCH1* (rarely reported in AML), *BCOR/BCORL1*, or *RUNX1* genes (25). Interestingly, MK AML (as well as CK AML) are commonly associated with a catastrophic mutational phenomenon, namely, chromothripsis, that is promoted by clustered genomic rearrangements that result in multiple oncogenic hits and tumor-suppressors’ inactivation (26). Eventually, such genomic events lead to the development of a highly proliferative disease. Specific aberrations involving chromosomes 3, 5, and 7 also clustered within HR-AML category. While chromosomes 5 and 7 aberrations are a common cytogenetic feature of trAML (4) and sAML that developed from previous myelodysplasia or myeloproliferative neoplasms (27), abnormalities involving chromosome 3 [inv(3)(q21.3q26.2) or t(3;3)(q21.3;q26.2); *GATA2,MECOM(EVI1*)] could be related to a distinct (usually *de novo*) clinical-biological entity (2, 28). *TP53* mutations and aberrant RAS pathway activity (*NRAS, KRAS, PTPN11, NF1*) are common features of trAML (27), as well as of *EVI1*-rearranged (*EVI-r*) AML (28, 29). Importantly, the latter is characterized by typical morphologic features (dysplastic megakaryocytes, multilineage dysplasia, and normal/elevated blood platelet counts) and driven by distinct molecular programs (like *MECOM* and *IKZF1*). *EVI-r* AML is associated with very poor overall survival (OS) (28, 29).

Furthermore, the 2017 European LeukemiaNet (ELN) adverse-risk category (2) also comprises specific WHO-defined genetic entities, which include wild-type *NPM1* and *FLT3-*ITD^high^, mutated *RUNX1*, and mutated *ASXL1*. The molecular pathogenesis of each distinct genetic entity is very poorly understood, and future studies are needed to investigate such biological complexity.

### Ongoing Adoptive Cell Therapies

The ability of leukemic blasts to evade immune surveillance has been recognized as a major mechanism of leukemia relapse after allo-HSCT (30). The novel use of immune therapeutics that aims to redirect the immune system against malignant blasts is now considered a new powerful tool to eradicate leukemia. Indeed, novel cellular immunotherapies with chimeric antigen receptor (CAR) T cells for B-lymphoproliferative disorders have recently achieved promising results (31) and generated great enthusiasm in the scientific community. As a matter of fact, a great number of studies are now emerging with the goal to provide similar effective treatments for various hematologic neoplasms. Cell therapy for AML is more complex than for lymphoid malignancies because myeloid leukemia-specific targets still need to be well identified. Many adoptive cell strategies are under investigation (**Table 1**) and are object of several clinical trials (45).

**Table 1:** **|** Selected published studies of immune cell-based strategies other than allogeneic transplantation for high-risk acute myeloid leukemia (HR-AML).

Type of immune cell-based therapy	Study design	AML patient cohort included in the study	Outcomes	ClinicalTrials.gov or others identifier
**Natural killer (NK) adoptive immunotherapy**	Prospective trial of NK cells from haploidentical KIR-ligand–mismatched donors after fludarabine/cyclophosphamide chemotherapy, followed by IL2.	17 adult acute myeloid leukemia (AML) patients (pts) in CR1^a^	CR^b^ = 9/16 pts (56%). (1 patient died due to infection).	NCT00799799 (32)
			Follow-up duration: 6–68 months.	
	Phase 1 non-randomized open-label, dose-escalation trial of CNDO-109-Activated allogeneic NK Cells.	12 adult AML pts in CR1^a^ not eligible for allo-HSCT^c^ and at high risk for disease recurrence.	CR^b^ = 3/12 pts (25%).	NCT01520558 (33)
			Follow-up duration: 32.6–47.6 months.	
	Phase 1 dose-escalation trial of membrane-bound interleukin 21 (mb-IL21) expanded donor NK cells infused before/after haploidentical allo-HSCT^c^.	8 adult high-risk AML pts in morphologic remission.	CR^b^ = 7/8 pts (88%).	NCT01904136 (34)
			Follow-up duration: 7.9–15.9 months.	
	Phase 2 trial of donor NK lymphocyte infusion (NK-DLI) after haploidentical allo-HSCT^c^.	8 pediatric and adult AML pts of a cohort of 16 pts with high-risk leukemia and highly malignant solid tumors.	Relapse rate = 4/8 pts (50%).	NCT01386619 (35)
			CR^b^ rate and follow-up duration were not specifically detailed for AML cohort.	
**Cytokine-induced killer (CIK) cells**	Phase I study of allo-CIK cells in pts with blood tumors relapsed after allo-HSCT^c^.	4 adult AML pts.	Response in 0/4 pts (0%).	N/A^f^ (36)
	Prospective enrolling study of allo-CIK in pts with high-risk leukemias relapsed after cord-blood transplantation.	4 adult AML pts, including 2/4 R/R^e^ AML and 2/4 in CR2^d^.	PR^g^ in 1/4 pts (25%).	N/A^f^ (37)
			Follow-up duration: ~4 months.	
	Retrospective study of allo-CIK administered after allo-HSCT^c^ in pts with high-risk leukemias.	5 adult AML pts (n=5).	CMR^h^ = 4/5 pts (80%).	N/A^f^ (38)
			Follow-up duration: 6.9–16 months.	
	Phase I/II clinical trial of autologous CIK in pts with AML.	13 adult AML pts in CR^b^.	CR^b^ = 6/13 (46%).	NCT00394381 (39)
			Follow-up duration: 38–50 months.	
**Chimeric antigen T (CART) cells**	Phase I study of autologous CAR anti-LeY T-cell therapy for AML.	4 adult R/R^e^ AML pts, including 3 pts treated in cytogenetic minimal residual disease, and 1 pt in progressive disease.	CR^b^ = 1/4 (25%).	CTX 08-0002 (40) (Australia)
			Follow-up duration: 23 months.	
	Phase I/II study of autologous CD33-directed CAR-T cells (CART-33) for the treatment of R/R^e^ AML.	1 adult AML pt.	Partial remission (PR) = 1/1.	NCT01864902 (41)
			Follow-up duration: 3 months.	
	Interventional open-label pilot study of RNA-redirected anti-CD123 autologous T-cell in patients with R/R^e^ AML.	5 adult AML pts.	CR^b^ = 0/5.	NCT02623582 (42)
			All patient progressed at day 28.	
	Single-center phase I dose-escalation study of a single infusion of autologous NKG2D-CART cells without lymphodepleting conditioning in subjects with AML.	7 adult AML pts, including 3 with CK, 3 with *TP53* mutation, and 4 secondary AML.	No objective response.	NCT02203825 (43)
**Chimeric antigen natural killer (CAR-NK) cells**	Phase I study of CD33-CAR NK-92 cells in R/R^e^ AML pts.	2 adult and 1 adolescent AML pts.	2/3 pts achieved CR^b^.	NCT02944162 (44)
			Relapse occurred in the 2 pts, ~4 months after CAR-NK cells infusion	

a CR1, first complete remission; ^b^CR, complete remission; ^c^Allo-HSCT, allogeneic hematopoietic stem cell transplantation; ^d^CR2, second complete remission; ^e^R/R, relapsed/refractory; ^f^N/A, not available; ^g^PR, partial response; ^h^CMR, complete molecular remission; ^i^CK, complex karyotype.

#### Natural Killer (NK) Cells Adoptive Immunotherapies

NK-cells are a subset of peripheral blood lymphocytes that are innately able to kill malignant cells through different mechanisms based on the balance between activatory and inhibitory signals. Interaction between major histocompatibility complex class I (MHC-I) molecules with killer immunoglobulin receptors (KIRs) on NK cells plays a major role in regulating NK cell function and activity. NK-cells kill leukemia cells when a mismatch between KIRs and their ligand on target cells is present (46). Such activity was demonstrated in T cell-depleted HLA-haploidentical transplant setting by the Perugia group and referred to as “NK cell alloreactivity” (47). The absence of any sort of pharmacologic immune suppression in TCD haplo-HSCT allowed for leukemia killing by alloreactive NK cells. On the other hand, the use of conventional immune suppressives to prevent GvHD in other transplant platforms may limit NK cell alloreactivity and its clinical effect (48). Further, NK cells may be dysfunctional and fail to kill AML blasts in case of abnormal phenotype, decreased degranulation level, and low INF-gamma and TNF-alfa production (32, 49, 50). For many years, NK cell adoptive transfer has been investigated as a possible approach to treat HR-AML. Studies showed donor-derived allogeneic NK-cells achieved durable complete remission in ~33% of HR-AML patients. Such studies proved infusion of a high number of NK-cells to be safe and well-tolerated. Indeed, donor NK cells appear not to cause any GvHD (33–35, 51). Moreover, donor NK-cells are able to persist and expand *in vivo* after infusion. On the other hand, while promising, allogeneic NK cell adoptive transfer has still limited efficacy, with generally low overall response rate. To overcome such limitations and boost NK cell *in vivo* function, different protocols and schemes that aim to generate and activate NK-cells are under evaluation and further studies are needed to establish the most effective approach.

#### Cytokine-Induced Killer (CIK) Cells

CIK cells derived from cytotoxic T lymphocytes (CTL) that are *in vitro* activated by anti-CD3, OKT3, INF-gamma and subsequently expanded with IL-2. Other than T cell markers, they express surface protein similar to NK-cells, such as CD56, the inhibitory NK receptors, and the natural killer group 2 member D (NKG2D) receptor, one of the most important receptors involved in NK-mediated cancer cell killing (45). In clinical trials, CIK cells have been generated both from autologous and allogeneic lymphocytes and have been infused in combination or not with different strategies of allo-HSCT. Even if the results of early trials were disappointing (37), last studies are more encouraging (36, 38, 39, 52, 53). CIK cell transfer resulted in stable complete remission in ~60% of patients with AML. No significant infusion-related toxicities and a very low rate of acute GvHD were observed after CIK cell infusions. No studies focused on HR-AML, so that the efficacy in this setting still remains to be determined.

#### Chimeric Antigen Receptor (CAR) T and NK Cells

CAR-T cells are genetically engineered T cells to express a variable heavy and light chains (V_HL_) on cell surface with high specificity for malignant cell antigens (54). Despite the great enthusiasm that followed the CAR-T cell success in the treatment of acute B-lymphoblastic leukemia/lymphoma (B-ALL/LBL) and forms of B-cell lymphoma, generation of CAR-T cells against myeloid leukemic blasts is challenging because of the absence of leukemia-specific target antigens. In fact, AML antigens are often widely expressed by other hemopoietic cells or tissues. While *in vitro* studies and xenografts demonstrated the effectiveness of anti-CD33 and anti-CD123 CAR-T cells (42, 55), clinical efficacy on AML is still to be confirmed. CD33 is a transmembrane receptor expressed on >90% of blasts, but unfortunately also on multilineage hematopoietic progenitors and myelomonocytic precursors. It was still validated as therapeutic target based on the efficacy of gemtuzumab ozogamicin, a drug-conjugated monoclonal antibody against CD33. Preliminary data of anti-CD33 CAR-T cells are not encouraging (41). CD123 is a transmembrane subunit of the IL-3 receptor expressed on 100% of AML cells, and its expression is increased in FLT3-mutated AML. *In vitro* ed *in vivo* (xenograft) preliminary data showed an increased cytokine release and decreased tumor burden using anti-CD123 CAR-T cells. FLT3 receptor is typically expressed on myeloid blasts, independent of FLT3 mutational status. Anti-FLT3 CAR-T cells showed *in vitro* ed *in vivo* promising antileukemic effect (56, 57). Moreover, these seem to be less toxic on normal hematopoiesis than the anti-CD33 counterpart. Many other potential targets are now under evaluation. Lewis antigen (LeY) is overexpressed on myeloid blasts in comparison to normal tissues. A trial of autologous CAR-T cells targeting LeY showed a biological response (~60% of patients), but relapse occurred within 2 years (40). An ongoing clinical trial (NCT03222674) evaluates the feasibility, safety, and efficacy of multi-CAR-T cell therapy that targets different AML surface antigens (Muc1/CLL1/CD33/CD38/CD56/CD123) in patients with relapsed/refractory AML. Another phase I study (NCT04156256) evaluates the safety and tolerability of CD123-CD33 dual CAR-T in patients with relapsed and/or refractory AMLs.

In alternative to CAR-T cells, CAR-NK cell therapy ideally combines the specific targeting provided by CARs with the NK cell ability to kill AML blasts in the absence of relevant systemic toxicity. Indeed, CAR-NK cells showed promising results with no important toxicity in lymphoma patients (58). Mouse preclinical models suggest that CD123 CAR-NK cells may be effective in AML (59). Clinical-grade CAR-NK cells can be manufactured from multiple sources (e.g., peripheral blood mononuclear cells, umbilical cord blood, hematopoietic progenitors, induced pluripotent stem cells), including the recently introduced CAR NK-92 cells, which consist of a modified CAR-engineered form of the NK-92 cell line. Such cell line represents an easily manageable and cost-effective tool for large-scale production of CAR-NK cells. Conversely, few drawbacks should be taken into account when using such strategy for CAR-NK cell manufacturing: i) failure of an *in vivo* expansion, due to lethal irradiation before infusion; ii) lack of NK-cell activating molecules (CD16 and NKp44); iii) potential *in vivo* tumorigenicity (60). The first-in-human clinical trial using CD33 CAR-NK cells derived from engineered NK-92 cells on three relapse/refractory extramedullary AML patients had no encouraging results (1/3 reached a transitory complete remission of 4 months) (44). Other trials with CD33 CAR-NK cells are under investigation (NCT02892695, NCT02944162). Such studies will help to clarify whether combinatorial strategies can provide antileukemic activity in the absence of relevant toxicity. While there was no specific focus on HR-AML in these preliminary studies and no clear studies showed HR-AML to be particularly sensitive to immune killing, the development of an effective anti-AML CAR-T or CAR-NK cell approach might provide a potent tool for reducing relapse in this high-risk disease.

#### Other Adoptive Cell Therapies in the Near Future

T cells can be engineered to target different tumor-associated antigens that are frequently expressed in advanced AML blasts and other hematological neoplasms. Preliminary results of such tumor**-**associated antigen lymphocyte therapy (TAA-**T)** for different relapsed hematologic malignancies after allo-HSCT (11 patient, Hodgkin’s lymphoma n=2, B-ALL n=3, AML n=5, and 1 HR-AML post 2^nd^ allo-HSCT) showed that 80% of patients (4/5) with AML achieved a stable complete remission (61). This study also suggested TAA-T to be safe and tolerable (only one patient showed a liver GvHD; no cytokine release syndrome or neurotoxicity was observed). These preliminary promising data suggest that TAA-T therapy may be a feasible option for preemptive treatment of relapse after allo-HSCT for HR-AML, but further clinical studies are needed to ascertain its feasibility and efficacy in this setting. T-cell receptor (TCR)-modified T-cell therapy is a novel emerging strategy using the anti-tumor effect of genetically modifying T cells through the transduction of TCR genes against several cancer antigens (62, 63). The impact of this therapy against specific leukemic antigens is still under investigation. This therapy seems also very safe (64). The *in vitro* and *in vivo* preliminary studies on B-malignancies are very promising (65).

### Allo-HSCT Strategies: Is There Room for the Cure of High-Risk Acute Myeloid Leukemias?

Allo-HSCT is the only treatment modality that can provide a long-term survival benefit for HR-AML (**Table 2**), although current conventional transplantation strategies have scarce effect on HR-AML outcomes, with a maximum 2-year OS of 30–35% and a higher relapse rate when compared to other cytogenetic risk categories (14–16).

**Table 2:** Selected published strategies of allogeneic hematopoietic stem cell transplantation (allo-HSCT) for high-risk acute myeloid leukemia (HR-AML).

Allo-HSCT strategy	Study design	AML patient cohort in the study	Conditioning regimen	Graft-*versus*-Host Disease (GvHD) prophylaxis	Outcomes	Ref.
**HLA-matched allo-HSCT**	Retrospective multicenter study of URD^a^ and MSD^b^ allo-HSCT in patients (pts) with high-risk acute myeloid leukemia (HR-AML) in CR1^c^.	584 adult HR-AML pts:	MAC^e^:	ATG^g^:	3-year OS°:	(66)
		- CK^d^: 32%	- MSD^b^: n=183	- MSD^b^: n=18	- MSD^b^=45%	
		- -7/del(7q): 25%	- URD^a^: n=252	- URD^a^: n=96	- HLA-well-matched URD^a^= 37%	
		- Others: 43%	RIC^f^:	CsA^h^:	- Partially-matched URD^a^=31%	
			- MSD^b^: n=252	- MSD^b^: n=155	Median follow-up:	
			- URD^a^: n=106	- URD^a^: n=137	- MSD^b^: 61 months	
				Tacrolimus:	- URD^a^: 35 months	
				- MSD^b^: n=40	3-year TRM^i^:	
				- URD^a^: n=191	- MSD^b^=21%	
				T-cell depletion:	- HLA-well-matched URD^a^=26%	
				- MSD^b^: n=20	- Partially-matched URD^a^=47%	
				- URD^a^: n=29		
				Others/missing:		
				- MSD^b^ n=11		
				- URD^a^: n=		
	Retrospective multicenter study of MSD^b^, MUD^p^, and MMUD^q^ allo-HSCT in CK^d^ AML pts.	1,342 adult CK^d^ AML pts:	MAC^e^: n=739	T-cell depletion: n=665	2-year OS^m^ = 36.8%	(14)
		- 357 with -7/del(7q)	RIC^f^: n=603		2-year NRM° = 17.6%	
		- 259 with -5/del(5q)				
**HLA-haploidentical allo-HSCT**	Prospective multicenter trial of G-CSF-primed grafts for haploidentical allo-HSCT in pts with blood neoplasms.	45 adult AML pts:	MAC^e^: n=64	ATG^g^	18-month LFS^x^ = 44%	(67)
**(Haplo-HSCT)**		- 34 standard-risk AML	RIC^f^: n=16	CsA^h^		
		- 11 HR-AML		Methotrexate		
		In HR-AML group:		Mycophenolate		
		- 2 pts in CR3^n^		Basiliximab		
		- 9 pts with active disease				
	Retrospective multicenter study of unmanipulated haploidentical allo-HSCT in patients with AML.	Within the entire AML cohort:	MAC^e^	CsA^h^	4-year OS^h^ = 57%	(68)
		- 99 pts in CR^l^		Mycophenolate		
		- 51 pts with active disease150 adult AML pts:				
		- 95 HR-AML				
	Retrospective single-center analysis of MSD^b^ *vs* URD^a^ *vs* HRD^r^ allo-HSCT for pts >60 years with AML.	94 adult AML pts:	In HRD^r^ allo-HSCT:	In HRD^r^ allo-HSCT:	4-year TRM^i^ = 20%	(69)
		- 28 HR-AML	MAC^e^: n=0	Post-transplant cyclophosphamide	2-year OS^h^ = 55%	
		Within the entire AML cohort:	Non-MAC^e^: n=9	CsA^h^	2-year TRM^d^ = 24%	
		- 80 pts in CR^l^	RIC^f^: n=24	Mycophenolate	2-year GRFS^e^ = 32%	
		- 14 with active disease				
	Prospective trial of TCR^s^ HRD^r^ allo-HSCT in pts with blood neoplasms, compared with a retrospective cohort of pts treated with TCD^t^ haplo-HSCT.	65 pts:	TCR^s^ group (n=32):	In TCR:	1-year OS^m:^	(70)
		- 42 AML/MDS	- MAC^e^: n=26	Post-transplant cyclophosphamide, Tacrolimus,	- TCR^s^ = 64%	
			- RIC^f^: n=6	Mycophenolate	- TCD^t^= 30%	
			TCD^t^ group (n=33):	In TCD:	1-year TRM^i^:	
			- MAC^e^	ATG^g^	- TCR^s^ = 16%	
					- TCD^t^ = 42%	
	Prospective trial of α/β TCD^t^ HRD^r^ allo-HSCT without ATG in children with chemorefractory AML.	22 AML:	MAC^e^	Bortezomib and tocilizumab +/− abatacept	2-year OS^m^ = 53%	(71)
		- 9 HR-AML			2-year EFS^v^ for HR-AML = 44%	
		- 10 primary refractory			TRM^i^ = 9%	
		- 12 R/R^u^ AML with active disease				
	Retrospective analysis in children with HR-AML in CR^l^ receiving α/β TCD^t^ HRD^r^ allo-HSCT or MUD^p^.	73 HR-AML:	MAC^e^	36 pts ATG^g^, tacrolimus and methotrexate	3-year OS^m^: 74%	(72)
		- 59 pts in CR1^c^		47 pts ATG^g^, Bortezomib and rituximab	OS^m^:	
		- 14 pts ≥ CR2^w^			- MUD^p^ = 64%	
					- haplo-HSCT = 86%	
					GRFS^z^:	
					- MUD^p^ = 49%	
					- haplo-HSCT = 70%	
					TRM^i^:	
					- MUD^p^ = 14%	
					- haplo-HSCT = 5%	
	Prospective trial of α/β TCD^t^ and B cell-depleted HRD^r^ allo-HSCT in children with AL.	80 AL:	MAC^e^	ATG^g^	For entire cohort:	(73)
		- 24 CR^i^ (CR1^c^=16, CR2^w^=8)			5-year OS^m^ = 72%	
		- 4 HR-AML			5-year CRFS^y^ = 71%	
					5-year TRM^i^ = 5%	
					For AML sub-cohort:	
					5-year LFS^x^ = 68%	
	Retrospective multicenter comparative analysis of URD^a^- or α/β TCD^t^ HRD^r^ allo-HSCT in children with AL.	342 AL:	MAC^e^	In HRD^r^ allo-HSCT:	For α/β TCD^t^ haplo-HSCT AL cohort:	(74)
		- MUD^p^: 127		α/β^+^ and CD19^+^ negative selection + ATG^g^	5-year probability of OS^m^ = 68%	
		- MMUD^q^: 118			5-year LFS^x^ = 62%	
		- HRD^r^: 98			5-year CRFS^y^ = 59%	
		105 CR^l^ AML:			TRM^i^ = 9%	
		- MUD^p^: 43			Cumulative incidence of relapse for AML sub-cohort = 21%	
		- MMUD^q^: 32				
		- haplo-HSCT: 30				
	Prospective single-arm clinical trial of naïve TCD^t^ peripheral blood stem cells grafts for adult pts with high-risk leukemia.	35 Adult high-risk leukemia:	MAC^e^	Tacrolimus	2-year OS^m^ = 78%	(75)
		- 10 AML				
	Prospective single-center trial of adult AML pts undergoing HRD^r^ allo-HSCT combined with regulatory and conventional T cells adoptive immunotherapy	50 adult AML pts:	Age-adapted MAC^e^	None	29-month OS^m^ = 77%	(76)
		- 20 HR-AML			CRFS^y^ = 75%	
		- 42 CR^i^			CRFS^y^ (for HR-AML) = 72%	
		- 8 with active disease			TRM^i^ = 21%	
	Cumulative Incidence of relapse: 4%					

a URD, unrelated donor; ^b^MSD, matched sibling donor; ^c^CR1, first complete remission; ^d^CK, complex karyotype; ^e^MAC, Myeloablative conditioning regimen; ^f^RIC, Reduced-intensity conditioning regimen; ^g^ATG, anti-thymocyte immunoglobulin; ^h^CsA, cyclosporin; ^i^TRM, transplant-related mortality; ^l^CR, complete remission; ^m^OS, overall survival; ^n^CR3, third complete remission; ^o^NRM, non-relapse mortality; ^p^MUD, matched unrelated donors; ^q^MMUD, mismatched unrelated donors; ^r^HRD, haploidentical related donor; ^s^TCR, T-cell replete; ^t^TCD, T-cell deplete; ^u^R/R, relapsed/refractory; ^v^EFS, event-free survival; ^z^GRFS, GvHD-free, relapse-free survival; ^w^CR2, second complete remission; ^X^LFS, leukemia-free survival; ^y^CRFS, chronic GvHD-​free, relapse-free survival.

#### HLA-Matched Allo-HSCT

A multicenter study of HLA-matched allo-HSCT that employed various immunosuppressive strategies for GvHD prophylaxis (cyclosporine A, tacrolimus, and T-cell depletion) compared a total of 584 patients carrying HR-AML in first complete remission (CR1) from 151 transplantation centers. It showed a median 3-year OS of 45% (range 38–52%), 37% (range 31–44%), and 31% (range 22–41%) in patients undergoing matched sibling donor (MSD), HLA-well-matched and partially-matched unrelated donor (MUD) transplantation, respectively. Myeloablative or reduced-intensity conditioning (RIC) regimens were used. Cumulative incidence (CI) of relapse at 3 years was 37% for MSD, 40% for well-MUD, and 24% for partially-MUD, while 3-year relapse-free survival (RFS) was 42, 34, and 29%, respectively. No significant differences in relapse were observed among the various cytogenetic subsets (66). Another retrospective multicenter study that involved more than 500 transplantation centers reported outcomes of 1,342 patients with CK-AML. Increased risk of relapse correlated with age, secondary AML, active disease at transplant, and the presence of deletion/monosomy 5. High tumor burden before transplant negatively impacted on post-transplantation outcomes. Indeed, 2-year CI of relapse for patients in CR and with active disease at transplantation was 47 and 64%, respectively. A very short OS at 2 years post-transplantation was observed in a subgroup of patients carrying deletion or monosomy 7 and deletion or monosomy 5 (29 and 20% respectively *vs* 42% in control groups without 7 and 5 deletion/monosomy). No significant survival benefit was observed between fully myeloablative conditioning and RIC regimen for patients with CK AML (34 and 28%). RFS rate was 39.9, 33, and 18.3% for patients ages <40, 40 to 60, and >60 years, respectively (14). Such studies demonstrate that the high relapse incidence after transplant in HR-AML patients is the major limitation of the procedure. Such outcomes urge the development of novel transplantation approaches.

#### Haplo-HSCT

The recent advancements in T-cell manipulation and in GvHD prophylaxis make haplo-HSCT a valuable transplantation strategy to overcome intrinsic chemotherapy resistance of high-risk leukemias. Haplo-HSCT procedures can be mainly divided in two major categories: T-cell depleted (TCD) peripheral-blood stem cells (PBSCs) haplo-HSCT and unmanipulated haplo-HSCT.

Unmanipulated haplo-HSCT relies on pharmacologic GvHD prophylaxis, and it is now adopted worldwide. The use of G-CSF-primed grafts (67, 77), post-transplant high-dose cyclophosphamide (PT-Cy) in combination with other immunosuppressive drugs (78, 79), and post-transplant rapamycin (80), are different approaches that have been tested in this setting. While such strategies help to keep non-relapse mortality (NRM) acceptable, disease relapse remains a major concern, especially when non-myeloablative conditioning regimens are used (81). A study of unmanipulated G-CSF-primed haplo-HSCT showed a 1-year CI of NRM of 36% and a CI of relapse of 21% at 1 year and 28% at 5 years respectively, with a 3-year probability of OS and RFS in 44 and 30%, respectively, in high-risk patients (> second CR or active disease) with hematologic malignancies (including HR-AML) (67). Haplo-HSCT with PT-Cy is now the most widely adopted haplo-HSCT platform, thanks to acceptable rates of acute and chronic GvHD, low NRM, no need of graft manipulation and contained costs. On the other hand, relapse rates are still disappointing in HR-AML patients. In fact, subanalyses showing outcomes of patients with adverse genetic risk AML reported relapse rates up to 50% (14). Because of such limitation and with the goal of reducing leukemia relapse, high-intensity myeloablative conditioning regimens have been employed. *Chiusolo P et al.* (68) and *Devillier R et al.* (69) showed a CI of AML relapse of 24% at 4 years and 25% at 2 years, respectively. Further studies will be needed to evaluate if such strategies are effective in subsets of HR-AML patients.

#### T-Cell Depleted Haplo-HSCT

In the last 20 years, several strategies of *ex vivo* T-cell depletion (TCD) have been tested to improve outcomes of acute leukemia patients who underwent haplo-HSCT. While traditional TCD procedure based on positive selection of CD34+ cells was associated with delayed immune reconstitution and increased risk of NRM (70, 82), more recent strategies are directed towards the preservation of immune subsets that improve post-transplant immune recovery for more effective anti-infective and antileukemic activities (82). Among these, αβ T-cell-depleted haplo-HSCT appears to be an effective platform for the treatment of HR-AML. In αβ T-cell-depleted haplo-HSCT the graft is manipulated to eliminate T cells that express αβ T cell receptor and which are demonstrated to be the main T cell population responsible for alloreactions that cause GvHD. In the studies by *Shelikhova L et al.* (71) and *Maschan M et al.* (72), children with primary refractory or relapsed AML who underwent αβ T-cell-depleted haplo-HSCT reached hematologic complete remission, despite 9/22 of them carried adverse-risk cytogenetics. However, the relapse rate and OS at 2 years after allo-HSCT were 42 and 52%, respectively. In different studies by *Locatelli F et al.* (73) and *Bertaina A et al.* (74), αβ T-cell and B-cell-depleted haplo-HSCT proved to be a safe and suitable approach in high-risk acute leukemias (HR-AL) in children. Indeed, it achieved a 5-year probability of chronic GvHD-free/relapse-free (GRFS) survival of 71% in HR-AL patients (73). A novel TCD haplo-HCT platform employs grafts that have been selectively depleted of naive T-cells. Indeed, depletion of naïve T cells (T_N_) from PBSC preserves hematopoietic engraftment and allows for the transfer of donor-derived memory T cells, that can confer immunity against pathogens with low risk of GvHD (75). This approach has demonstrated to improve outcomes of HR-AL patients (the 2-year relapse rate was 21% and the 2-year RFS was 70%) in a single-arm trial (75). Thus, such approaches are promising, but relapse rates still reduce outcomes of HR-AL patients.

#### Haploidentical HSCT Combined With Regulatory and Conventional T-Cells Adoptive Immunotherapy

We have recently demonstrated that haplo-HSCT combined with regulatory and conventional T-cells adoptive immunotherapy (Treg-Tcon haplo-HSCT) is able to overcome disease-intrinsic chemoresistance (18, 76). We enrolled 50 AML patients in the study; 40% of them (20/50) had HR-AML. An “age-adapted” myeloablative conditioning based on total body irradiation (TBI) for patients up to the age of 50 years and total marrow/total lymphoid irradiation (TMLI) for patients aged 51–65 years was followed by thiotepa, fludarabine, and cyclophosphamide. No pharmacological GvHD prophylaxis was given. Two millions/kg donor regulatory T cells were given at day −4 to allow for their alloantigen-specific *in vivo* expansion. One million/kg conventional T cells were given at day −1 and were followed by the infusion of a “megadose” of purified CD34+ hematopoietic progenitor cells at day 0. Fifteen/50 patients developed grade ≥2 acute GvHD (aGvHD). Moderate/severe cGvHD occurred in only one patient. Only two patients relapsed (4%). Consequently, at a median follow-up of 29 months, the probability of moderate/severe cGvHD/relapse-free survival was 75% (18, 76). TMLI allowed to safely extend the powerful effect of a myeloablative conditioning to older (>60 years old) patients. Further, when looking at the different genetic signatures of the enrolled AML patients, we found that HR-AML did not have a higher risk of relapse in comparison to more favorable subgroups. Indeed 17 of the 20 HR-AML patients are alive and leukemia-free despite many of them had detectable disease at transplant. Such results demonstrate HR-AML to be sensible to immune-mediated killing. Indeed, the absence of pharmacologic immune suppression in Treg/Tcon haplo-HSCT could have favored a potent GvL effect that was exerted across all the AML subsets and that was not limited by disease burden and previous refractoriness to chemotherapeutic agents. ELN AML genetic risk stratification is considered to retain outcome prediction after allo-HSCT (83). However, our study showed that effect was lost after Treg/Tcon haplo-HSCT in a single series of 50 AML patients. While larger multicentric studies are needed to support such conclusion, the potent GvL activity of Treg/Tcon haplo-HSCT appears to be an effective tool for the treatment of such unfavorable AML.

## Discussion

HR-AMLs are usually characterized by a very poor response to conventional treatments and to conventional allo-HSCT. Indeed, relapse rates are high (often above 50%) and result in very low survival (often below 10–20%). Thus, novel effective strategies are needed. Recent studies on new adoptive cell strategies (CAR-T cells, CAR-NK cells, CIKs, activated NK cells) bring new hopes for the treatment of such unfavorable diseases. Indeed, immune-cell-based therapies may represent a powerful tool to successfully treat chemoresistant HR-AML. NK cell adoptive immunotherapies are a promising therapeutic, but their efficacy is still limited and fine-tuning of the approach is still required for larger clinical use (32–35). The more recently introduced CAR-T- and CAR-NK-cell-based treatments demonstrated high potency in pilot studies and hold great promise (40–44). The growing body of clinical studies and broader use of these agents in different settings and against novel targets will provide key information on their ability to eradicate HR-AML. Furthermore, we have recently demonstrated that Treg-Tcon haplo-HSCT is able to overcome HR-AML intrinsic chemoresistance, prevent relapse, and improve survival (18, 76). This study strongly suggests that HR-AMLs are sensitive to antileukemic immunity. The introduction of new immune therapeutics that strengthen immune activity against leukemia and the development of transplantation approaches that favor unopposed GvL might help to develop powerful tools for an effective treatment of HR-AML.

## Data Availability Statement

The original contributions presented in the study are included in the article/supplementary material. Further inquiries can be directed to the corresponding author.

## Ethics Statement

All procedures were in accordance with the ethical standards of the institutional research committee. The patients/participants provided their written informed consent to participate in this study.

## Author Contributions

RL and AMar contributed equally to the figures and writing. AMan, SB, and EH reviewed the manuscript. AV provided guidance, and AP wrote the manuscript and provided critical review. SH and LR provided critical review. All authors contributed to the article and approved the submitted version.

## Funding

These studies were supported by the grant from the “Associazione Italiana per la Ricerca sul Cancro (AIRC)”, START-UP Grant n. 20456 to AP.

## Conflict of Interest

The authors declare that the research was conducted in the absence of any commercial or financial relationships that could be construed as a potential conflict of interest.

## Publisher’s Note

All claims expressed in this article are solely those of the authors and do not necessarily represent those of their affiliated organizations, or those of the publisher, the editors and the reviewers. Any product that may be evaluated in this article, or claim that may be made by its manufacturer, is not guaranteed or endorsed by the publisher.
